# Ultrasound Monitoring of Extant Adnexal Masses in the Era of Type 1 and Type 2 Ovarian Cancers: Lessons Learned From Ovarian Cancer Screening Trials

**DOI:** 10.3390/diagnostics7020025

**Published:** 2017-04-28

**Authors:** Eleanor L. Ormsby, Edward J. Pavlik, John P. McGahan

**Affiliations:** 1 Department of Radiology, University of California Davis Medical Center, 4860 Y Street, Suite 3100, Sacramento, CA 95817, USA; jpmcgahan@ucdavis.edu; 2 Department of Radiology, Kaiser Permanente Sacramento, 2025 Morse Ave, CA 95825, USA; 3 Division of Gynecologic Oncology, Department of Obstetrics and Gynecology, University of Kentucky Chandler Medical Center-Markey Cancer Center, Lexington, KY 40536, USA; Epaul1@uky.edu

**Keywords:** ovary, cancer, screening, monitoring, surveillance, serial ultrasonography

## Abstract

Women that are positive for an ovarian abnormality in a clinical setting can have either a malignancy or a benign tumor with probability favoring the benign alternative. Accelerating the abnormality to surgery will result in a high number of unnecessary procedures that will place cost burdens on the individual and the health delivery system. Surveillance using serial ultrasonography is a reasonable alternative that can be used to discover if changes in the ovarian abnormality will occur that favor either a malignant or benign interpretation. Several ovarian cancer screening trials have had extensive experiences with changes in subclinical ovarian abnormalities in normal women that can define growth, stability or resolution and give some idea of the time frame over which changes occur. The present report examines these experiences and relates them to the current understanding of ovarian cancer ontology, presenting arguments related to the benefits of surveillance.

## 1. Introduction

Ovarian cancer is the deadliest cancer that women face, causing more deaths than any other cancer of the female reproductive system [1]. However, the prevalence of ovarian cancer is low, responsible for only about 3% of all cancers in women [2] and accounting for a lifetime risk of 1.3% (1 in 75) [3]. Transvaginal ultrasound (TVS) has been widely recognized as the first line for evaluating adnexal masses presenting both low risk and low cost. Prospective ovarian cancer screening trials have utilized TVS to detect early stage malignancies. The five-year survival rate for women diagnosed with stage I ovarian cancer has been reported to be as high as 95% [4,5] in contrast to only 30% for women with stage III disease [6]. While large prospective screening trials have focused on how best to identify malignancies in asymptomatic women in the general population, adnexal masses are commonly identified by ultrasound ordered for a wide variety of indications in routine clinical practice even when a patient does not present with relevant symptoms. While the US Preventive Services Task Force (USPSTF) has recommended against population screening for ovarian cancer [7], many women undergo ultrasound for various symptoms. This paper reviews recent prospective ovarian cancer screening trial findings for clinical application on how women with adnexal masses, found by ultrasound, for various reasons other than for screening purposes, should be managed and followed.

Ovarian cysts are often observed sonographically even in post-menopausal women with a reported incidence rate of up to 21% [8]. The question of how best to manage these masses has been the subject of much interest and debate among clinicians including obstetric gynecologists, primary care physicians, radiologists and gynecology oncologists. Several reports have asserted that resected ovarian cysts do not contain malignancy [9,10,11], but that if left unmonitored, ovarian cysts can progress to ovarian cancers [12,13]. Therefore, all ovarian cysts may present some source of concern. Historically, this concern has led to a conundrum among radiologists and clinicians. Should these cysts be monitored (how frequently and for how long) or should ovarian cysts be managed operatively at the risk of potential harm from surgical complications and medical expenses?

In 2010, a consensus panel of the Society of Radiologists in Ultrasound (SRU) that was composed of 19 experts in radiology, obstetric gynecology, and gynecology oncology, as well as pathology released a recommendation regarding the management of adnexal masses found sonographically in asymptomatic women [14]. The panel analyzed literature available at the time of the conference (October 2009) and strategies in clinical practice with the goal of reaching a consensus on: (1) which masses might not require follow-up, (2) which masses would need imaging follow-up, as well as when follow-up evaluation should occur, and (3) which masses should warrant referral to a gynecologic oncologist for surgical evaluation. The consensus agreed that it is reasonable to perform annual ultrasound follow-up of cysts larger than 5 cm in premenopausal women and those larger than 1 cm in postmenopausal women, although such cysts are unlikely to be malignant [14]. A recent expert review suggested that low risk abnormalities can undergo an initial three-month follow-up with those that remain stable or decreasing in size being examined every 12 months for five years [15].

Since the SRU guidelines from 2010 [14], differences over how best to manage adnexal masses persisted and were recently addressed by the first international consensus conference on adnexal masses [15]. This panel included representatives of societies in the fields of gynecology, gynecologic oncology, radiology and pathology and clinicians from Europe, Canada and the United States. While many of the adnexal masses are benign appearing (i.e., simple cysts or hemorrhagic cysts), for many more, it is not clear whether the mass may contain foci of malignancy and consequently are classified as *indeterminate*. As a clarification of terminology, “simple cysts” and “unilocular cysts” are the same and are characterized as being anechoic structures that are absent papillae, solid areas and septa (complete or incomplete). The low prevalence of ovarian cancer (3%) [2] establishes the likelihood that most ovarian cysts are benign yet cysts cannot be dismissed because they occur with a high incidence rate (21–35%) [8]. Some cysts are not simple and include morphologic elements that can demonstrate multiseptations or small solid nodules. No specific guideline had been established for indeterminate masses by the SRU consensus due to the fact that data analyzing long-term follow up of adnexal masses at the time was insufficient. The SRU stated that “as research continues, the recommendations regarding management of adnexal cysts may vary”. The present review examines the evidence from recent research in histopathology of ovarian cancer types, ovarian cancer screening trials and ultrasound morphology of adnexal masses to establish a framework for surveillance of these masses.

## 2. Type 1 and Type 2 Ovarian Cancers Found in Ultrasound Imaging

Currently, ovarian cancers now include two distinct types of malignancy: Type 1 or 2 based on histologic pathogenesis, molecular alterations and clinical progression (Table 1). Type 1 ovarian cancers include low grade serous carcinoma, endometrioid carcinoma, and clear cell carcinoma. Type 1 ovarian cancers demonstrate a step-wise progression originating from a benign precursor or borderline tumor or endometriosis [16,17,18]. For example, low grade serous carcinomas may arise via transformation of benign and borderline serous tumors that are thought to be derived from inclusion cysts originating from the ovarian surface or tubal epithelium. This progression is analogous to the adenoma-to-carcinoma sequence seen in colorectal carcinoma pathogenesis or the hyperplasia-to-carcinoma sequence in endometrioid carcinoma of the endometrium [19]. 

In contrast, Type 2 ovarian cancers are highly aggressive and include high grade serous, high grade endometrioid and undifferentiated carcinomas, as well as malignant mixed mesodermal carcinomas, usually presenting at an advanced stage [17,19,20]. Type 2 ovarian cancers often have TP53 mutations but rarely have mutations that are associated with Type 1 ovarian malignancies [17,20]. Some Type 2 ovarian cancers (in particular, high grade serous carcinoma) are associated with *BRCA* (BReast CAncer susceptibility gene) inactivation [21]. Compelling evidence indicates that these malignancies may originate from the epithelium of the fimbrial portion of the fallopian tube as serous tubal intraepithelial carcinomas (STIC) [22,23,24,25,26,27,28,29,30,31,32]. Finally, some high grade serous carcinomas have been reported to develop from transformation of serous borderline tumors or low grade Type 1 serous carcinomas [17,18,19,20]. While pathogenesis may differ, the morphology of the high-grade serous carcinomas that develop in the Type 2 pathway is similar to high-grade serous carcinomas that are transformed from Type 1 tumors with shared clinical behaviors [17]. Using this paradigm, a stratified treatment plan can be devised. However, currently there is no prospective means that differentiates between the subtypes of ovarian cancer based on ultrasound imaging. Based on recent ovarian cancer screening results, abnormalities with lesser degrees of morphologic complexity may harbor micro foci of ovarian cancer indicating that a wide spectrum of abnormal morphology should be considered for ultrasound follow up and active surveillance. 

### 2.1. Summary of Information from Recent Prospective Ovarian Cancer Screening Trials

There have been four large prospective ovarian cancer screening trials utilizing ultrasound in asymptomatic women [5,33,34,35]. The first randomized control trial in the US was the Prostate, Lung, Colorectal and Ovarian Cancer Screening (PLCO) Trial, a randomized controlled trial (RCT) of 68,616 women aged 55 to 74 of whom 30,630 underwent screening between 1993 and 2007 [34]. Women were screened using serum CA-125 (cancer antigen 125) at a cut-off of ≥35 kU/L and transvaginal ultrasound (TVS) for four years followed by CA-125 alone for an additional two years. Endpoint analysis showed that screening with the combination of CA-125 and transvaginal ultrasound had no mortality benefit compared to the unscreened control group [34]. Importantly, in the PLCO study, surgical decisions were made on the basis of a single ultrasound exam and an absolute CA-125 level of 35 units/mL. More importantly, the PLCO trial had no uniform evaluation and treatment algorithm for patients with screen-detected adnexal masses so that women identified in the screening arm could be treated up to nine months after ultrasound detection, allowing their disease to progress to later stages during this time.

In the multicenter prospective randomized Shizuoka Cohort Study of Ovarian Cancer Screening (SCSOCS) trial in Japan [33], conducted between 1985 and 1999, asymptomatic postmenopausal women were assigned either to a screening arm (*n* = 41,688) or to a control arm (*n* = 40,799). Furthermore, 63% of ovarian cancers detected by screening were stage I disease versus 38% in the control arm. Importantly, optimal tumor debulking was achieved more often in women whose ovarian cancer was detected by screening [33]. Assessment of ovarian cancer specific survival was not completed in the SCSOCS trial.

More recent studies have been published with a screening strategy that improves on using a single ultrasound exam or a single CA-125 value at 35 units/mL, an approach that did not achieve an acceptable positive predictive value (PPV) in the PLCO trial [34]. These strategies include the use of serial ultrasound instead of a single ultrasound exam dictating the surgical decision and the utilization of multimodalities keying on changes in serial CA 125 determinations. The University of Kentucky Ovarian Cancer Screening Trial utilized a prospective single arm that focused on annual ultrasound screening study of 25,327 women from 1987 to 2012 [36]. In the Kentucky study, serial ultrasound follow up of the 6807 women with ovarian abnormalities displaying varying ultrasonographic morphologic features resulted in a 304% improved PPV from 8.1% to 25% and reduced unnecessary surgery on benign tumors [36]. Importantly, this study found that women in the screening group had a higher rate of earlier stage cancer discovery (68% stage I or II disease) than the unscreened comparison group (27% stage I or II, *p* < 0.01) [36,37,38]. Overall five-year survival of women who had epithelial ovarian cancer (EOC) found during the serial ultrasound follow up including false negative cancers was 74.8% ± 6.6% compared to 53.7% ± 2.3% for women who were clinically detected (*p* < 0.01) [37,38]. Using the serial ultrasound approach, differentiating benign from malignant tumors was based on the regression of benign masses [36]. Extending serial ultrasound to include a quantitative index showed that malignant tumors demonstrated increasing morphology index scores over time [37,39].

Others have evaluated serial CA-125 level or other biomarkers such as human epididymis protein 4 (HE4) to improve the detection of ovarian cancer [40,41,42]. The Risk of Ovarian Cancer Algorithm (ROCA) is a multivariate linear model based on longitudinal data from women with ovarian cancer and estimates intermediate and high risk for malignancy based on changes in CA-125 levels relative to an individual’s previous levels. ROCA with multiple CA-125 determinations has performed better in detecting ovarian cancer than a single level since CA-125 levels vary greatly depending on the menopausal status, fertility drug use, current cigarette use, race, pelvic inflammation and irregular menstruation [43]. Using an absolute CA-125 cut off value of 35 units/mL may result in a high false negative rate because only 50–60% of women with stage 1 EOC will have CA-125 elevated above this level and borderline, and Type 1 or low grade tumors are known to express low levels of CA-125 [44].

In the United Kingdom Collaborative Trial of Ovarian Cancer Screening (UKCTOC), the largest randomized control screening trial to date, performed between 2001–2005, 202,638 women from the general population were assigned to a control group (no intervention) or to annual screening using either transvaginal ultrasound (USS) or serum CA-125 interpreted by ROCA with transvaginal ultrasound as a second line test (multimodal screening, MMS) [12,35,44]. The stage distribution of the screen-detected primary invasive cancers was similar in both the multimodality group and the group that received only ultrasonography [35]. In addition, 50% of primary invasive ovarian and tubal malignancies detected by serial ultrasound screening alone had stage I or II disease versus 26% in the control cases detected clinically (i.e., without screening) [35]. Screening produced a significant increase in the detection of early stage ovarian malignancy. A report on the survival benefit from the UKCTOCS has been published, which showed that, when prevalent cases were excluded, a significant mortality reduction was noted after 7–14 years within the multimodality arm [35]. Similar but lesser mortality reduction was seen with ultrasound alone. The trial is currently undergoing additional follow up to further examine mortality reduction. Based on these data, it was concluded that 641 screens are needed to prevent one ovarian cancer death [35].

Recently, it has been reported that ovarian cancer screening detects more indolent and less aggressive Type 1 cancers [45] and that the frequency of Type 2 cancer is ~75% is higher than Type 1 with higher mortality rate for Type 2 cancer due to its faster rate of growth and metastasis. This result is in contrast to findings from the Kentucky Ovarian Cancer Screening trial where 83.3% of early stage malignancies were aggressive Type 2 cancers [5,35,36,38]. In the UKCTOC ultrasound arm trial, both Type 1 and Type 2 cancers were detected albeit more Type 1 than Type 2 [35]. Of the 23 Type 2 cancers diagnosed in the UKCTOC ultrasound arm, 15 were associated with adnexal abnormalities, while eight had normal ultrasound with subsequent diagnosis of ovarian cancer within 16 months (ranging 6–13 months with median of 10) [12]. No women with persisting normal ultrasound results were found to have Type 1 ovarian cancers of the 32 women with Type 1 cancer who were detected by ultrasound in the ultrasound arm of the UKCTOC [12]. Based on these observations, it may be concluded that many Type 2 cancers are found in women brought to clinical practice by symptoms and that Type 2 cancers have been shown to be quite possible to find through ovarian cancer screening using ultrasonography. Therefore, serial ultrasound follow up of persistent masses may benefit women in clinical practice by discriminating lethal Type 2 ovarian cancers as well as by reducing unnecessary surgery in cases where complexity moderates or abnormalities resolve. 

### 2.2. Can Type 2 Ovarian Cancers Be Detected by Ultrasound? 

Using a growth model of serous cystadenocarcinoma (Type 2) based on retrospective analysis of *BRCA*_1_ carriers who had undergone prophylactic bilateral salpingo-oophorectomies (PBSOs), it was noted that high grade serous carcinoma likely spends approximately 4.3 years as histopathologically detectable but clinically occult early stage tumors [46]. This analysis also stated that more than 50% of serous carcinomas advanced to stage III/IV by the time they reached 3 cm in diameter. Assuming spherical shape, this would be a volume of 14 cm^3^ (note that the normal ovary is 10–20 cm^3^ and a walnut is 22 cm^3^). The report postulated that the tumor would double in volume every two and a half months so that, at best, ultrasound follow up may only lead to the detection of low volume high grade Type 2 cancers rather than early stage cases. However, early stage disease detected in the Kentucky Ovarian Screening Program was larger than postulated by this model (Stage I Type 2: 65.4 cm^3^ ± 27.6, 27, 4.1, 366, *n* = 13; Stage II Type 2: 131.1 cm^3^ ± 33.4, 95.8, 10, 351.4, *n* = 14 (mean ± SEM, median, min, max)) [5,37]. Thus, the prediction made by the model [46] that to achieve 50% sensitivity in detecting tumors before they advance to Stage III, an annual screen would need to detect tumors of 1.3 cm in diameter is inaccurate and not supported by empirical screening data. Other investigators modeling the levels of CA-125 associated with the smallest progressing ovarian cancers reported that these cancers could develop unnoticed for 10.1 years and presented the view that the largest tumor below the resolution of ultrasound (0.5 cm diameter) could progress to a detectable size (1.2–2.5 cm) in 1–2 years [47]. Based on this estimation [47] and the Kentucky findings summarized above, early stage Type 2 ovarian malignancies are well within the range of discovery by ultrasound. In the context of surveillance monitoring, it would seem that arbitrary cessation as suggested by one retrospective study [48] of ultrasound follow up of small complex adnexal masses, which are less than 6 cm at seven months would miss both small volume high grade Type 2 cancers and the indolent Type 1 tumors that can potentially progress to higher grade invasive cancer.

## 3. Risk of Ovarian Cancer When There Is an Adnexal Mass

Adapting the information from these prospective ovarian cancer screening trials to non-screening applications in day-to-day clinical practice needs consideration. The USPSTF has recommended against ultrasound exams for ovarian cancer screen in asymptomatic women [7] based on prior randomized prospective ovarian cancer trials that failed to show mortality benefits while focusing on the risk of unnecessary surgery with a small immediate complication rate or more long-term effects of premature menopause from oophorectomy such as bone density loss. However, women present clinically with a wide variety of indications including nonspecific symptoms, as well as more gynecologic symptoms such as vaginal bleeding, pelvic fullness or pain. Sometimes, women may be referred for follow up ultrasound on incidental abnormal findings from other diagnostic radiology exams such as CT that have been obtained for unrelated reasons. Women who had any adnexal mass had a much higher relative risk of developing ovarian cancer as observed in the UKCTOC trial, compared to women who had no adnexal mass [12]. The relative risk ratio for all EOC (Types 1 and 2) was 49.2 for women with a multilocular solid cyst and 38.4 for women with a solid mass when compared to women with normal ultrasound exams [12]. For the most deadly and aggressive ovarian cancers (Type 2), the relative risk was 31.3 for women with a multilocular cysts with solid components and 38.4 for women with a solid mass [12].

Even benign appearing unilocular and multilocular cysts without any solid elements have been reported to be associated with epithelial ovarian cancer. In the UKCTOC report, unilocular and multilocular cysts without any solid components had a relative risk for EOC within three years of 5.3 (95% CI (confidence interval) 1.9–15.2) and 6.8 (95% CI 1.9–22.9), respectively, compared to normal ultrasound exams [12]. Among the primary EOC detected in the UKCTOC ultrasound screening trial, 16% (nine out of 55) developed from unilocular cysts while 9% (five out of 55) developed from multilocular cysts within three years of an initial scan. Among the borderline tumor and Type 1 epithelial cancers, 16% (five out of 32) developed from unilocular cysts while 13% (four out of 32) developed from multilocular cysts [12]. In another series by a separate research group, 11% (4/35) of borderline tumors and 4% (1/24) of epithelial ovarian cancers were classified as unilocular cysts at ultrasound examination performed by an ultrasound expert in a tertiary referral center for gynecological ultrasound [49]. 

Valentin et al. noted in their cohort that the overall malignancy rate for unilocular cysts was 1% and was higher among postmenopausal women (2.76%) then premenopausal women (0.54%) [50]. While the rates were very low, the difference was statistically significant between the two age groups. The authors of the study noted that, upon pathologic inspection, seven of the 11 malignant cysts described as unilocular on ultrasounds were found to contain small papillary projections or solid components, which were not observed sonographically [50]. Careful scrutiny of ultrasound images was advocated because subjective error or ultrasound resolution may provide explanations for the failure to observe the papillary projections. While there are limitations to ultrasound, the degree to which these limitations contribute to ultrasound results is small as shown by high sensitivities (>80%) and high negative predictive values (>99%) [5,37,38].

### 3.1. The Risk Profile for Abnormal Ultrasound Findings

Among postmenopausal women in the general US population, the overall risk of ovarian cancer rises with age to a 9–13% lifetime risk [51]. Relative risk increases when symptoms are present for which a pelvic ultrasound is often performed in clinical practice, mostly because of pelvic pain. The great majority of women with symptoms alone do not have an ovarian malignancy. The majority of women with both symptoms and an ovarian abnormality on ultrasound also do not have a malignancy due to the low prevalence of ovarian cancer; however, women with symptoms have been found to have a higher prevalence of ovarian cancer than that reported for asymptomatic women in screening trials using ultrasonography [52,53,54]. Differences between screening trial pelvic ultrasound outcomes and those in clinical settings result because symptoms predominate in clinical settings. 

### 3.2. Benefit of Serial Ultrasound Follow-Up

**Figure 1:**
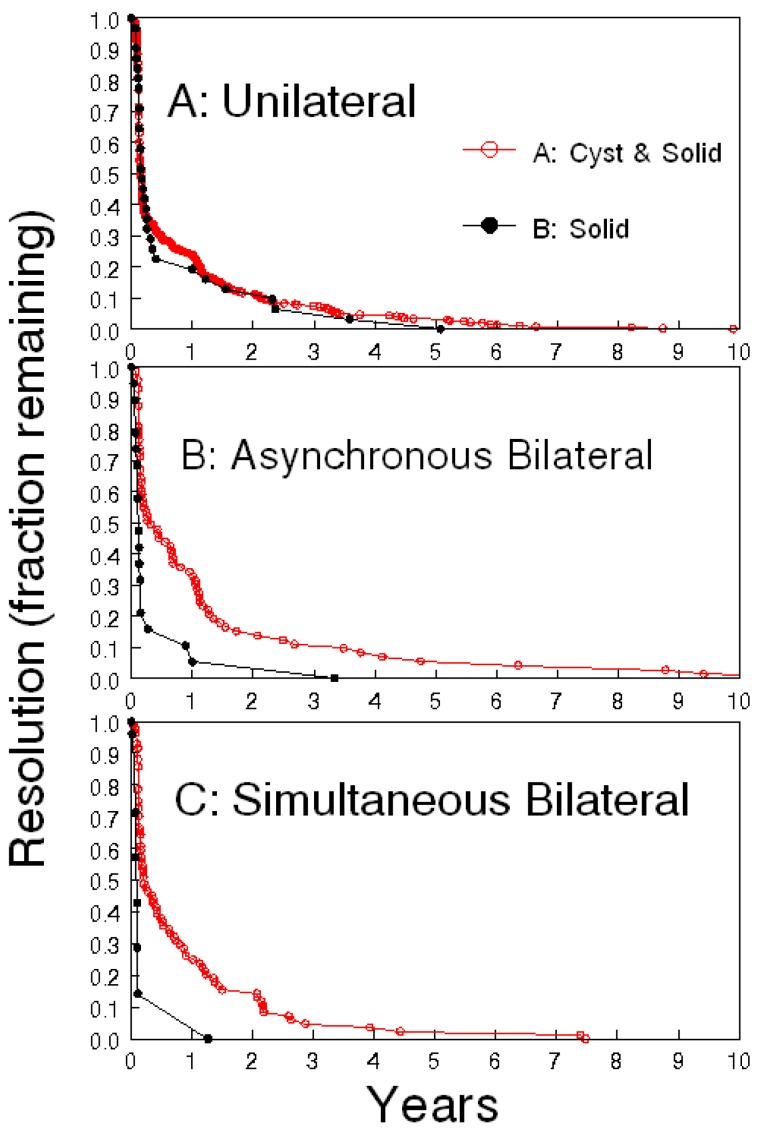
Resolution of complex ovarian abnormalities. (**A**) unilateral abnormalities, never simultaneously on both sides; (**B**) intermittent unilateral abnormalities consisting of ovarian abnormality on one side or the other at different times; (**C**) bilateral abnormalities occurring simultaneously on both sides. Cysts with solid components: red open circles. Solid components: black solid circles. Intrapanel comparisons, (**A**): not statistically different. (**B**) *p* < 0.001, (**C**) *p* < 0.001. Interpanel comparisons: **A** vs. **C** *p* < 0.01, **A** vs. **B** and **B** vs. **C**, not significantly different.

Serial ultrasound and a subsequent increase in morphologic complexity of an adnexal mass have been used as the basis for surgical decisions in the single arm trial at the University of Kentucky [37] and in the UKCTOC [35]. In the University of Kentucky trial, the majority of ovarian abnormalities resolved within a year with serial ultrasound, including indeterminate masses. More than half of women (63%) with ovarian cystic abnormalities had resolution in the subsequent follow-up with near exponential resolution of ovarian abnormalities so that, by 1–2 years, only a fraction of the ovarian abnormalities persisted (Figure 1, from [36]). 

Ovarian abnormalities that continue to persist comprise only a fraction of the ovarian abnormalities that are identified and are candidates for ongoing serial observation until their indeterminate status changes due to an increase in morphologic complexity. Therefore, serial ultrasound surveillance can mitigate the potential risk from surgical complications due to prematurely resecting indeterminate adnexal masses, especially if an adnexal mass demonstrates signs of resolving. Ultrasound follow-up is advantageous because it is cost effective and low risk. The cost of ultrasound follow-up is nominal compared to the cost of surgical treatment for women [55] and provides a greater margin of safety than dismissing an extant adnexal mass without follow-up based on presuming benign status due to an initial indeterminate ultrasound morphology.

## 4. Subjectivity

### 4.1. Does Stability Over Time Argue Against Malignancy?

**Figure 2:**
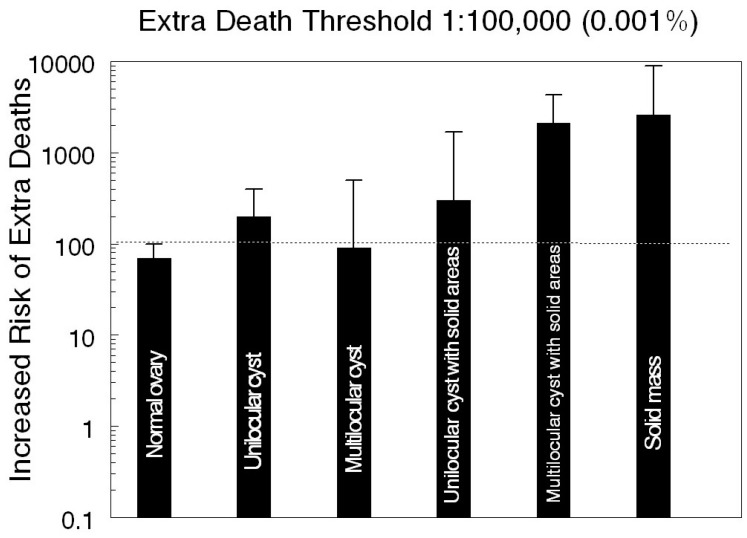
Estimation of risk in terms of extra deaths in women diagnosed with Type 2 primary epithelial ovarian cancer within three years after an ultrasound exam. Data were collected in the United Kingdom Collaborative Trial of Ovarian Cancer Screening Protocols as published [12] and normalized by the acceptable level of risk of no more than one extra death per 100,000 in environmental studies. Absolute risk of subsequent malignancy is shown by the bar labeled with each type of finding on the first ultrasound exam. The 95% confidence interval extends upward from each bar. The dashed line indicates the 95% confidence interval of the normal ovary extended across all types of findings.

To address this question, work that focused on the ultrasound discovery of adnexal masses was reviewed [13]. Malignancy has been found in stable masses, which enlarged and increased in morphologic complexity in up to three years after initial detection in the UKCTOCS [12]. To put the risk of prematurely terminating ultrasound surveillance in perspective, the definition of the acceptable risk level (ARL) from environmental studies [56] of no more than 1 extra death/100,000 was used to normalize the UKCTOCS trial data. Using this approach, the absolute risks for the appearance of malignancy in up to three years after an initial ultrasound exam as calculated from the UKCTOCS data [12] are considerably elevated (Figure 2). The risk of malignancy is higher after finding any of the ovarian ultrasound abnormalities as judged by the 95% CI (Figure 2). Even allowing the 0.001% ARL to be relaxed 10 fold would still lead to the expectation of a considerable number of extra malignancies within three years of the first scan. If prematurely stopping surveillance caused 50% or more of these malignancies to be diagnosed at an advanced stage, likely destined to be fatal, then extra deaths due to curtailing surveillance can be expected to be high and emphasizes the peril of limiting ultrasound surveillance [13].

### 4.2. The Conundrum of Ultrasound: Subjectivity and Technical Considerations

Subjectivity and operator-dependent errors are intrinsic to ultrasound imaging even when the images are acquired and interpreted by expert radiologists or gynecologists and contain subtle features that can go unreported or be missed. While the term *expert sonographer* is in wide use, there is no definition that provides an understanding of this status or terminology. Ultrasounds are very often performed by technologists whose varying skills and expertise are acquired and honed in the practice in which they are employed. For experts and technologists alike, small lesions can be missed due to various technical factors such as subject motion, lack of patient cooperation, large body habitus with poor acoustic penetration, bowel gas shadowing which obscures pelvic organs, positioning of the ovarian structure behind the uterus, etc. For some large masses, complete visualization of the wall and internal morphology cannot be obtained because the signal from the transvaginal probe cannot adequately reach the entire mass. When this is the case, the SRU recommendations advocate pelvic magnetic resonance images (MRIs) for better characterization and full visualization of large masses [14]. Small papillary projections within unilocular cysts can be absent on ultrasound, but later confirmed by surgical pathology. Thus, there can be situations where information from ultrasound can be inadequate.

Although ultrasound is highly sensitive, subjectivity inherent to the interpretation of ultrasound images accounts for variation in ultrasound reports especially for indeterminate adnexal masses. Recently, the International Ovarian Tumor Analysis (IOTA) study showed that there is considerable uncertainly and inter-observer disagreement when solid components and papillary projection were present [57]. Most disagreement was on the definition of a papillary projection, but there was also uncertainty leading to disagreement about whether a certain structure should be classified as a solid component or as a collection of septa, a collection of small cysts or as ovarian stroma. Including Doppler imaging can introduce variability because some septa can only be visualized with Doppler and, therefore it can change the type of morphology that is reported.

In addition to physiological cysts, serous and mucinous cystadenomas, transitional and germ cell tumors, struma ovarii, stromal cell tumors, fibromas, endometriomas, low malignant potential (borderline) tumors, and malignancies, and other structures that are expected to have the potential to be reported as having solid components in ultrasound exams of the adnexa include: inflammations, infections and abscesses. Only after surgery has been performed is it possible to establish the histopathologic identity of an ovarian abnormality seen on ultrasound. Histopathological identification is not a possibility in serial ultrasound surveillance when solid structures resolve as has been reported in the Kentucky study [36]. In brief, this study reported that while cysts with solid components had the highest risk for epithelial ovarian cancer, many complex abnormalities (cysts with apparent solid areas) and apparent solid masses were more likely to resolve within a year of surveillance (76.5–80.6%) than unilocular cysts and cysts with septations (32.8–43.9%, *p* < 0.001) [36]. Complex abnormalities and solid masses had a median time to resolution of 7.8–8.7 weeks, while unilocular cysts and cysts with septations had a median time to resolution of 53–55.6 weeks. The expectation is that if these were truly solid masses that are highly suspicious for cancer, they should not resolve. There are several possibilities to explain this observation. First, something other than the ovary was measured in the ultrasound report (i.e., overlapping adjacent tissue like a bowel loop). Second, the plane through which a partially solid ovarian structure was sonographically examined exaggerated the extent to which the structure appeared to be solid. Third, unverified factors like inflammation, infection or abscess were responsible for reporting solid areas in the ultrasound report, providing pseudo-findings. Serial ultrasonography provides a protection against a pseudo-finding of solid structure whenever there is evidence of a resolving process or resolution. Few would argue that uncertainty can be eliminated in ultrasound exams, especially with subjective interpretation providing the foundation for what is reported. The degree to which subjective interpretation can account for the identification of apparently “solid components” that subsequently resolve is not presently known, but can be corrected by a serial ultrasound imaging approach in diagnostic imaging. Moreover, the utilization of complementary Doppler imaging could contribute to differentiating a truly solid mass as distinct from a mass of clotted blood. However, even with Doppler imaging, not all solid masses will be able to demonstrate Doppler flow if there is too much tissue for the ultrasound beam to penetrate or if certain tumors are not sufficiently vascularized for detection by Doppler imaging. Thus, in the absence of definitive Doppler identification, the best solution for distinguishing apparently solid components is serial ultrasonography.

## 5. Ovarian Mass Ultrasound Morphology

**Figure 3:**
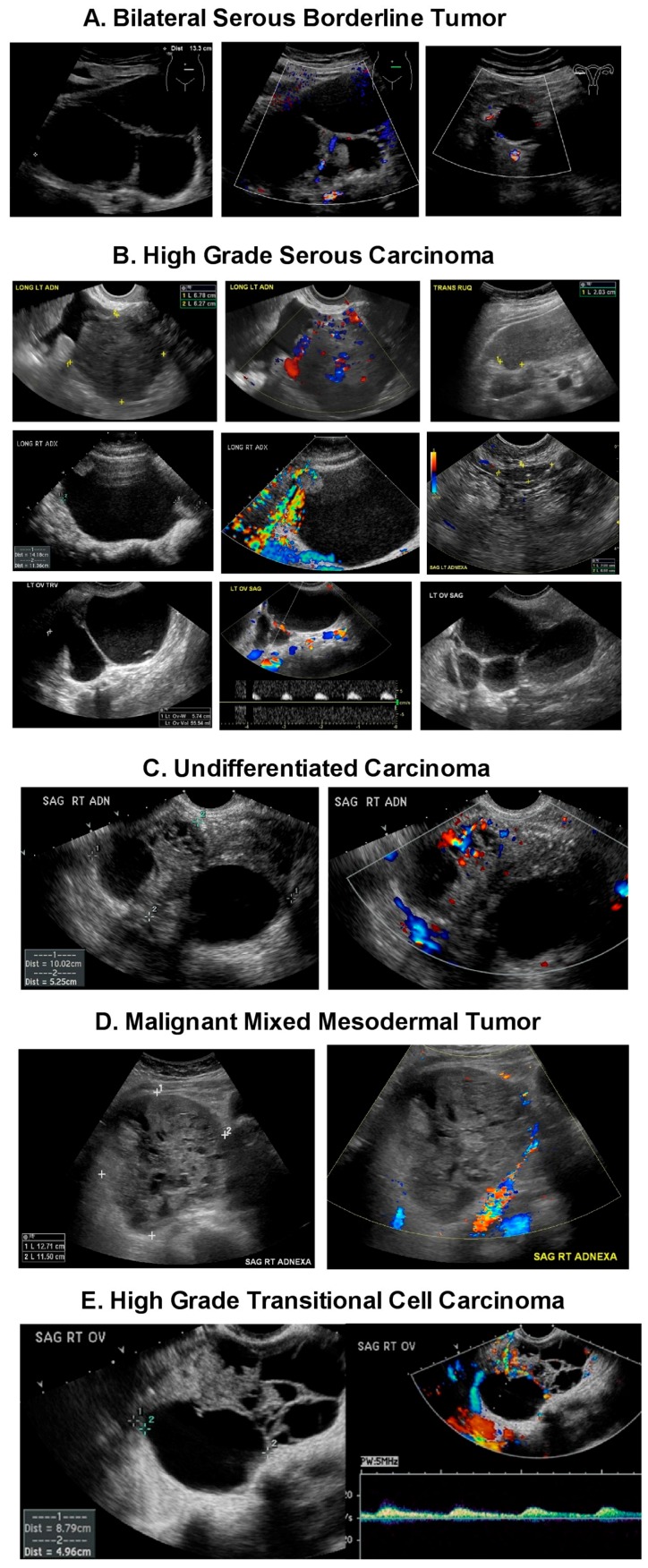

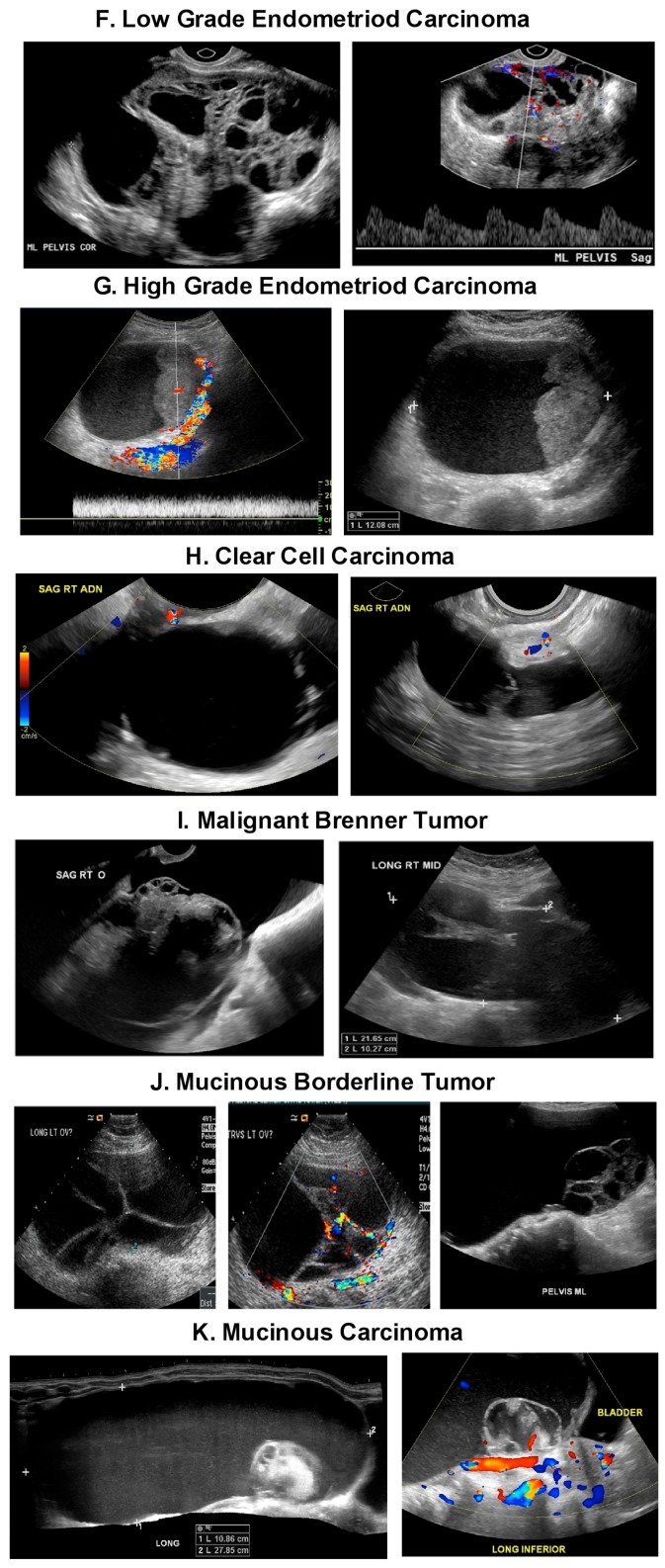
Ultrasonographic appearance of borderline, Type 1 and Type 2 ovarian cancers. (**A**) Bilateral Serous Borderline Tumor: tumors of low malignant potential (i.e., borderline tumors) account for 15% of all epithelial ovarian cancers. Nearly 75% of these tumors are stage I at the time of diagnosis. They represent a heterogeneous group and occur in younger women with favorable prognosis. However, symptomatic recurrence and death may be found as long as 20 years after therapy in some patients. (**B**) High Grade Serous Carcinoma (Type 2): serous carcinomas comprise the majority of ovarian carcinomas. Unlike low-grade serous carcinoma, *TP53* mutation occurs in up to 80% of high-grade tumors [17,20]. (**C**) Undifferentiated Carcinoma (Type 2): about 5% of ovarian cancers are so poorly differentiated and difficult to classify that they are called undifferentiated carcinomas and occur as large, solid hemorrhagic structures with necrosis. (**D**) Malignant Mixed Mesodermal Tumor (Type 2): occur almost exclusively in postmenopausal women. (**E**) High grade transitional cell carcinoma (Type 2) is probably not a distinct entity but a poorly differentiated form of serous or endometrioid carcinoma. (**F**) Low Grade Endometrioid Carcinoma (Type 1): endometriosis a likely precursor of endometrioid carcinoma. (**G**) High grade Endometriod carcinoma (Type 2) is morphologically indistinguishable from high grade serous carcinoma. (**H**) Clear Cell Carcinoma (Type 1): as with endometrioid carcinomas, there is a close association between endometriosis and clear cell carcinoma. (**I**) Malignant Brenner Tumor (Type 1): relatively uncommon neoplasm. Most Brenner tumors are benign, only 2–5% being malignant. (**J**) Mucinous Borderline Tumor (Type 1): 53.3% of borderline tumors are serous tumors and 42.5% are mucinous tumors (42.5%). (**K**) Mucinous Carcinoma (Type 1): frequently has a heterogeneous composition with coexisting elements of cystadenoma, stromal microinvasion, noninvasive carcinoma, and invasive carcinoma.

**Figure 4:**
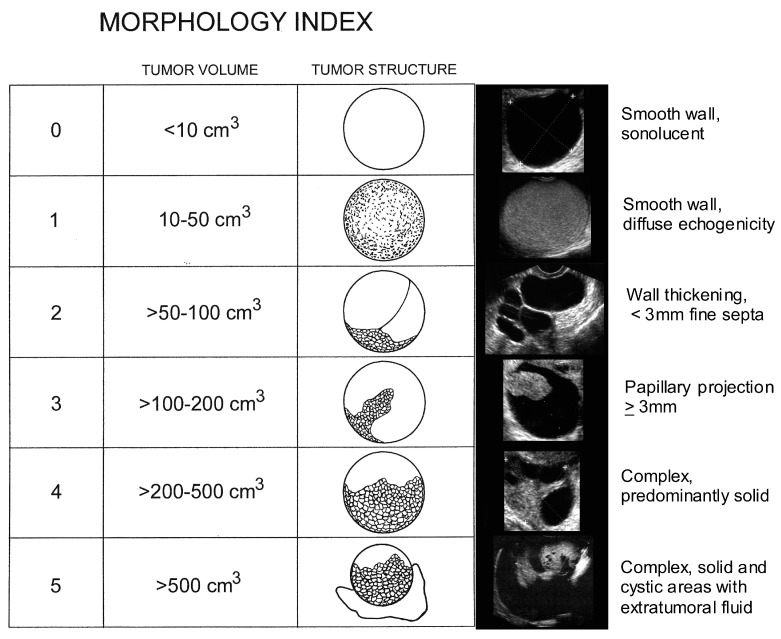
Morphology Index evaluation of ovarian abnormalities. Part of the figure is reprinted from [39,58].

There is considerable overlap between the ultrasonographic morphology of ovarian masses. In the UKCTOCS study, 25 (78.1%) of the borderline/Type 1 cancers had adnexal abnormalities with solid elements (unilocular solid/multilocular solid cysts or solid masses) on the initial (*n* = 23) or subsequent (*n* = 2) scans [12]. Of the 23 women diagnosed with Type 2 EOC, 15 had sonographic adnexal abnormalities where eleven (47.8%) had solid elements or ascites on the initial scan [12]. While in the UKCTOCS study, the strongest association between ovarian morphology and epithelial ovarian cancer was the presence of “solid component(s)”, borderline, and Type 1 and Type 2 cancers were found across all sonographic morphologies including unilocular and multilocular cysts without solid components. In contrast, benign pathology was the norm for all morphologies including cysts with solid components [36]. The challenge for radiologists and gynecologic oncologists is correctly diagnosing epithelial ovarian cancers associated with indeterminate masses having multiple thick septations and or solid components that can be seen across borderline, indolent Type 1 tumors, aggressive Type 2 tumors and benign masses. This challenge is complicated by the low prevalence of ovarian cancer. Clear expressions of ovarian abnormalities seen ultrasonographically are presented in Figure 3. Tumors of low malignant potential (i.e., borderline tumors) account for 15% of all epithelial ovarian cancers (Figure 3A). Nearly 75% of these tumors are stage I at the time of diagnosis. They represent a heterogeneous group and occur in younger women with favorable prognosis. However, symptomatic recurrence and death may be found as long as 20 years after therapy in some patients. While low grade serous tumors (Type 1) occur less frequently, pernicious high-grade serous carcinomas (Type 2) predominate, accounting for over half of ovarian malignancies, Figure 3B. Undifferentiated carcinomas (Figure 3C, 2%), malignant mixed mesodermal tumors (Figure 3D, 3%) and high grade transitional cell carcinomas (Figure 3E, 2%) (all Type 2) each carry a serious prognosis, but together account for less than 10% of ovarian malignancies. Endometriod carcinomas comprise ~20% of ovarian malignancies with low and high grade endometriod carcinomas appearing ultrasonographically similar (Figure 3F,G). Together with clear cell carcinomas (Figure 3H, 3%), malignant Brenner’s tumor (Figure 3I, <1%) and mucinous carcinomas (Figure 3J,K, 5%) are recognized as being responsive to treatment. Overlapping morphological components characterize all of these tumors. To discriminate malignant from benign abnormalities, a Morphology Index (MI) has been developed at the University of Kentucky [58]. The MI grades an abnormality on the basis of both size and structure (morphology) as shown in Figure 4. Increasing MI scores correlate well with the risk of an abnormality being malignant [39].

### 5.1. Malignant Degeneration of Benign Masses

It is well known that epithelial ovarian carcinomas can develop from ovarian endometriosis [59,60,61,62,63]. The strongest association is seen with endometrioid and clear cell carcinomas [64,65,66], which have been reported to be associated with ovarian endometriosis in 30–40% and 40–70% of cases, respectively [66,67]. Endometrioid cancer is considered as a Type 1 tumor while clear cell carcinoma is a more intermediate type [16]. Twenty-eight per cent of benign and 38% of borderline endometrioid tumors were reported to be associated with endometriosis in one series [68,69]. Thus, there are benign entities that can become malignant.

### 5.2. Psychosocial Elements in Prospective Ovarian Cancer Screening Trials

In an age when patients can freely review their medical charts, including their entire radiology report, and access the Internet for information, we enter uncharted territory in how to communicate our findings with patients. The cost in following an ovarian mass by ultrasound is nominal compared to surgery or extensive chemo-radiation treatment when ovarian cancer is detected at a later stage. When women were polled about screening for ovarian cancer by the University of Kentucky Ovarian Cancer trial team, 97% of the women surveyed reported that they wanted to be screened and that they would even pay for screening themselves because ovarian cancer has a mortality ratio that is four times greater than breast cancer, despite an incidence rate that is low [70] even with potential complications that range from long-term physiological changes such as bone density loss to surgical mortality.

It is legitimate to consider if serial ultrasound and surveillance impacts psychosocial well-being. Non-physical or psychological harm to women has been examined in the Kentucky Ovarian Screening trial. When compared to an age and education matched group with no history of ovarian screening, women in the Kentucky trial had more ovarian cancer-specific distress/anxiety, less optimism, and less knowledge about risk factors upon entry [71]. Thus, some distress or anxiety relative to ovarian cancer appears to play a motivating role for entering the Kentucky screening trial. As part of these efforts, the validity of self-reporting by women in the Kentucky trial was evaluated and found to be very high [72]. In a study with baseline, two-week and four-month measurement, recipients of a normal ovarian screening exam showed decreased ovarian cancer-related distress, increased positive effects and increased knowledge of risk factors [73], indicating, for the vast majority of women screened, that there are beneficial effects on ovarian cancer-specific anxiety, attitude and knowledge. Women who received an abnormal TVS screening result were found to have an elevated ovarian cancer-specific distress (but not general distress) at a two-week follow-up that returned to baseline at the four-month follow-up [74]. Results were influenced by a monitoring coping style, low optimism and family history of ovarian cancer. Needs that have been identified in women with an abnormal TVS screening result deal with anticipation, emotional responses, role of the sonographer and impact of prior cancer experiences [75]. In examining social cognitive processing vs. cognitive social health processing after an abnormal TVS screening, analyses found that greater distress was associated with greater social constraint [76]. Thus, psychological conditions that are apparently associated with ovarian screening are governed by different underlying factors in different women and not the screening result per se. Furthermore, recent published findings from the UKCTOCS data showed that screening does not necessarily provoke an unacceptable level of anxiety or psychological morbidity [77]. Taken together, these results support the position that surveillance and serial ultrasonography may not negatively impact perceptions of well-being, particularly if more women were made aware that some tumors may be low grade and slow growing.

## 6. Executive Summary of What We Already Know

There has been significant advancement in our understanding of ovarian cancer since the first randomized prospective ovarian cancer screen trials were initiated to detect cancers in early stages to reduce the mortality of this disease. We now know that ovarian cancer is a large heterogeneous group consisting of Type 1 (indolent and low grade tumor) and Type 2 (aggressive and high grade tumor) based on molecular, genetic make-up of the cancer and how they progress based on their precursors or genetic predisposition [16,17,18,19,20,21,22,23,24,25,26,27,28,29,30,31,32]. The evidence indicates that surgical treatment based on limited imaging or tumor marker data based on single or short-term exams has led to unnecessary surgery with potential for morbidity or mortality [34]. Ultrasounds in ovarian cancer screening have detected both Type 1 and Type 2 cancers even at early stages [5,12,35,36,37,38]. Because benign and malignant ovarian neoplasms share overlapping ultrasound morphologies, accounting for a high ratio of benign to malignant surgical findings and because ovarian cancer prevalence is low while the prevalence of ovarian abnormalities is high, active ultrasonographic surveillance of ovarian abnormalities based on the morphologic index provides the best means for detecting Type 2 ovarian cancers. Theoretical modeling on how Type 2 cancers behave has shown that it may be possible to detect low volume high grade cancer with better outcomes utilizing close follow-up with ultrasounds [46,47]. Ovarian cancer screening with ultrasound has detected a stage shift that finds malignancies at an earlier stage and serial ultrasound has increased the positive predictive value of this approach while decreasing false positive cases [5,36,37,38]. Medical-legal risk may enter the consideration when an indeterminate mass is not followed, often leading to surgery that proves unnecessary. Unnecessary surgery on false positive cases can have serious immediate complication rates ranging from 2–15% [12,34], but, if serial ultrasound indicates that the abnormality is resolving, then the need for surgery could be circumvented. Based on a comprehensive review of the literature, it can be concluded that:

(1) there are benefits in ultrasound monitoring of persisting indeterminate masses;
(2) resolution of sonographic abnormality defines benign status;
(3) stability over time may not equate with benign status particularly for Type 1 tumors;
(4) for certain types of tumors benign lesions are precursors of malignant lesions;
(5) repeated ultrasound monitoring does not negatively impact psychosocial well-being.

## 7. Conclusions

In conclusion, ultrasounds are inexpensive, associated with low morbidity, widely available, have high sensitivity in detecting abnormalities and are free of risk in image acquisition. Decisions for following ovarian masses detected by ultrasound in day-to-day practice differ from decisions for annual ovarian cancer screening in asymptomatic women with normal risk. The goal of ovarian cancer screening is to detect early stage ovarian cancer with improved mortality benefit. The role of ultrasounds in adnexal mass management should be to increase positive predictive value of detecting ovarian cancer to minimize unnecessary surgeries and to avoid failures to detect ovarian cancers. Findings from ovarian cancer screening trials and advances in our understanding of ovarian cancer pathogenesis can guide the management of adnexal masses found in clinical practice, especially since screening studies have observed that women with ovarian masses found by ultrasounds have a higher risk for ovarian cancer than those women who do not have an ovarian mass. Serial ultrasound surveillance using a morphologic index allows quantitative surveillance and the ability to distinguish benign masses based upon stable index scores (absence of growth, stable morphology) or decreasing index scores (resolution), while increasing index scores are strongly linked to malignancy. Concomitant use of serial CA-125 as in the ROCA model should also increase the positive predictive value of detecting malignancy. All improvements should promote a close working relationship between diagnostic radiology and clinicians using standardized structured reporting models as advocated by the American College of Radiology as seen in the Breast Imaging Reporting Data System (BI-RADS) or the Liver Imaging Reporting Data System (LI-RADS) to reduce ambiguous terminology, decrease variability in interpretation and improve communication.

## Figures and Tables

**Table 1:** Summary of Type 1 and Type 2 ovarian carcinomas.

Tumor Type	Type 1 Tumors	Type 2 Tumors
Behavior	Indolent	Aggressive
Diagnosis at	Early Stage	Advanced Stage
Survival Rate at 5 years	About 55%	About 30%
Type/Precursor	-Endometrioid carcinoma/Endometriosis -Clear cell carcinoma/Endometriosis Mucinous carcinoma/Mucinous Cystadenoma, Endometriosis, Teratoma, -Brenner Tumor, and Mucinous borderline tumor -Low grade serous carcinoma/Serous cystadenoma, Adenofibroma, Atypical proliferative serous tumor, Mullerian epithelial cyst -Transitional cell carcinoma or Malignant Brenner tumor/Brenner tumor	-High grade serous carcinoma/Probably de novo starting at the tubo, ovarian surface epithelium, serous tubal intraepithelial carcinomas (STIC) or ovarian hilum stem cell -Undifferentiated carcinoma? -Malignant mixed carcinoma?
